# Usefulness of EEG Techniques in Distinguishing Frontotemporal Dementia from Alzheimer's Disease and Other Dementias

**DOI:** 10.1155/2018/6581490

**Published:** 2018-09-03

**Authors:** Raffaele Nardone, Luca Sebastianelli, Viviana Versace, Leopold Saltuari, Piergiorgio Lochner, Vanessa Frey, Stefan Golaszewski, Francesco Brigo, Eugen Trinka, Yvonne Höller

**Affiliations:** ^1^Department of Neurology, Franz Tappeiner Hospital, Merano, Italy; ^2^Department of Neurology, Christian Doppler Klinik, Paracelsus Medical University, Salzburg, Austria; ^3^Karl Landsteiner Institut für Neurorehabilitation und Raumfahrtneurologie, Salzburg, Austria; ^4^Department of Neurorehabilitation, Hospital of Vipiteno, Vipiteno, Italy; ^5^Research Department for Neurorehabilitation South Tyrol, Bolzano, Italy; ^6^Department of Neurology, Hochzirl Hospital, Zirl, Austria; ^7^Department of Neurology, Saarland University Medical Center, Homburg, Germany; ^8^Department of Neurosciences, Biomedicine, and Movement Sciences, University of Verona, Verona, Italy; ^9^Centre for Cognitive Neuroscience, Paracelsus Medical University, Salzburg, Austria; ^10^University for Medical Informatics and Health Technology (UMIT), Hall in Tirol, Austria

## Abstract

The clinical distinction of frontotemporal dementia (FTD) and Alzheimer's disease (AD) may be difficult. In this narrative review we summarize and discuss the most relevant electroencephalography (EEG) studies which have been applied to demented patients with the aim of distinguishing the various types of cognitive impairment. EEG studies revealed that patients at an early stage of FTD or AD displayed different patterns in the cortical localization of oscillatory activity across different frequency bands and in functional connectivity. Both classical EEG spectral analysis and EEG topography analysis are able to differentiate the different dementias at group level. The combination of standardized low-resolution brain electromagnetic tomography (sLORETA) and power parameters seems to improve the sensitivity, but spectral and connectivity biomarkers able to differentiate single patients have not yet been identified. The promising EEG findings should be replicated in larger studies, but could represent an additional useful, noninvasive, and reproducible diagnostic tool for clinical practice.

## 1. Introduction

Alzheimer's disease (AD) and frontotemporal dementia (FTD) are the most common causes of dementia. However, the differential diagnosis is challenging due to their overlapping clinical symptoms and involved brain regions. Current clinical criteria identify distinct phenotypes of FTD on the basis of presenting clinical features; these include the behavioral variant of FTD (bvFTD), the agrammatic variant of primary progressive aphasia, and the semantic variant of primary progressive aphasia [1, 2]. The bvFTD is characterized by changes in social behavior and conduct, with loss of social awareness, poor impulse control, hyperorality, and dietary changes, as well as apathy and impaired performance in executive tasks [3]. Even if diagnostic criteria exist [4], the disease remains poorly recognized. Other less frequent causes of dementia, such as dementia with Lewy bodies (DLB) and Parkinson's disease dementia (PDD), are probably still underdiagnosed in the clinical setting, and also sometimes difficult to differentiate from AD and FTD.

Some neuroimaging techniques, such as positron emission tomography (PET), single photon emission computerized tomography (SPECT), and functional magnetic resonance (MRI) have been used in order to identify affected brain regions in dementias and to improve diagnostic accuracy. However, these imaging tools are invasive, expensive, and often not clinically feasible.

In contrast, the electroencephalogram (EEG) represents a noninvasive technique that is cheap, highly available, and sensitive to changes in the functional state of the human brain.

We aimed in this narrative review to summarize and discuss the most relevant studies dealing with EEG techniques in order to distinguish FTD from AD, other dementias, and healthy subjects.

## 2. Methods

The MEDLINE, accessed by PubMed (1966–February 2018) and EMBASE (1980–February 2018) electronic databases, was searched using the following medical subject headings (MeSH) and free terms: “Frontotemporal dementia,” “Frontotemporal lobar degeneration,” “Alzheimer's disease,” “Dementia with Lewy bodies,” “Lewy body dementia,” “Parkinson's disease dementia,” “Electroencephalography,” “Spectral analysis,” and “Connectivity.”

Only original articles written in English were considered eligible for inclusion. Review articles were excluded. For the selected titles, full-text articles were retrieved and their reference lists were searched for additional publications. In the case of missing or incomplete data, principal investigators of included trials were contacted and additional information requested. The titles and abstracts of the initially identified studies were screened to determine if they satisfied the selection criteria. Two reviewers independently assessed the methodological quality of each study and risk of bias, focusing on blinding. The search strategy described above yielded 10 results, two of which were excluded after reading the full paper, thereby leaving 8 studies which contributed to this review.

## 3. Electroencephalography (EEG)

### 3.1. EEG Analysis

The EEG represents an old and inexpensive method that has been employed for many years in dementia research. EEG has been examined in demented patients in order to differentiate individuals with various types and severity of cognitive impairment from healthy subjects. While visual EEG analysis still prevails in routine clinical practice, the differential diagnosis of the various subtypes of dementia relies on quantitative EEG (qEEG), where extensive technical knowledge is needed in the field of digital signal processing. One of the most frequently used research methods is the spectral analysis, and therefore sometimes the term qEEG is used to indicate quantitative spectral analysis. However, qEEG offers a wider spectrum of possible applications. By means of computational algorithms, such as fast Fourier transform (FFT) or autoregressive (AR) models [5–10], the characteristics of the EEG can be documented in an objective and quantitative way. Moreover, the EEG analysis is not restricted to the predefined surface localizations of the international electrode systems such as the 10-20 system. Advanced methods of EEG analysis have been applied to the study of neural activity sources in 3-D models of the brain, and different techniques known as solutions for the EEG inverse problem have been proposed throughout the years [11–14]. Furthermore, low-resolution brain electromagnetic tomography (LORETA) allowing 3-D localization of cortical EEG generators both in the time and frequency domains [15, 16], has been successfully applied to study EEG changes across normal elderly, mild cognitive impairment, and dementia [17–19]. In addition, standardized LORETA (sLORETA) [12], allows obtaining images of standardized current density with the so-called “0 localization error”.

### 3.2. EEG Studies

Generalized EEG slowing has been observed in a number of studies in AD during rest. This slowing can thus be assessed visually by qualitative EEG assessment as decreased frequency of the dominant background rhythm, or by spectral analysis as increased power of slow rhythms (*δ* and *θ* frequency bands) and reduced power of faster rhythms (upper *α* and *β* bands) [10, 20, 21]. Indeed, the peak frequency in the power spectrum, which is normally located between 8 and 12 Hz, shifts in AD to a lower range of 6–8 Hz. However, only a few studies have investigated EEG changes in FTD. Qualitative evaluation of EEG recordings typically shows no abnormal slowing in FTD patients [22]. We could like to point out that pathological slowing of the EEG can be seen as a more extreme form of the general slowing of the background rhythm that can be found also in healthy ageing. Thus, age-matched control groups are a necessary prerequisite for these studies; otherwise, the EEG-slowing effect will be overestimated.

**Figure 1:**
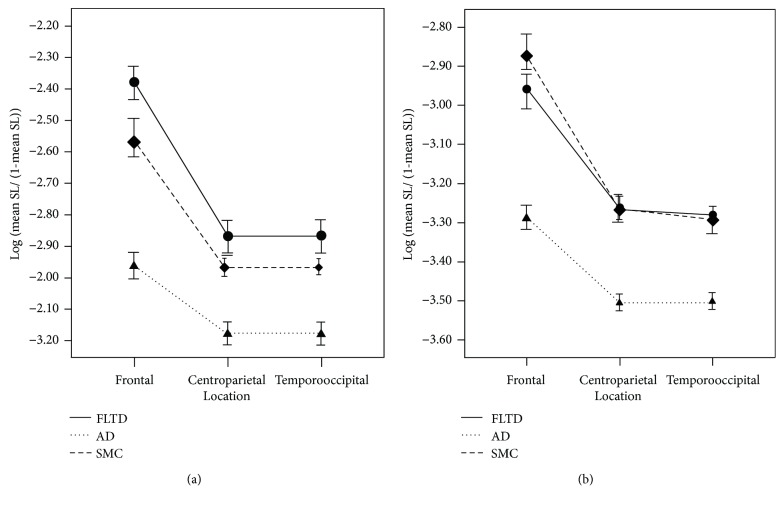
Significant group × electrode interaction effects in the 8–10 Hz frequency band (a) and the 10–13 Hz frequency band (b). Error bars indicate standard deviations. Legend: FTLD = frontotemporal lobar degeneration; AD = Alzheimer's disease; SMC = subjective memory complaints. Reproduced with permission from Pijnenburg et al. [23], in 2008.

Mild to moderate FTD and AD patients have been compared with healthy controls (HC) using a so-called visual grand total EEG score and the synchronization likelihood as a measure of functional connectivity [23]. No significant differences were found in the visual grand total EEG score between FTD and HC. AD patients show significant EEG slowing and a loss of reactivity compared with FTD and HC by means of the visual grand total EEG. AD patients exhibit decreased synchronization likelihood compared with both FTD and HC in fast frequencies, whereas no differences can be found between FTD and HC (Figure 1). Thus, the changes in synchronization likelihood parallel the pattern of slowing. The characteristics of the higher frequencies—be it power or synchronization—are reduced in AD, but not in FTD.

Several studies have investigated differences in qEEG among patients with FTD and those with AD, PDD, and DLB. For qEEG, the global field power was calculated for six frequency bands: *δ* (1.0–3.5 Hz), *θ* (4.0–7.5 Hz), *α* (8.0–11.0 Hz), *β*1 (12.0–15.5 Hz), *β*2 (16.0–19.5 Hz), and *β*3 (20.0–23.5 Hz). The spectral ratio was calculated as the ratio of the sum of fast frequency bands *α* + *β*1 + *β*2 + *β*3 and slow frequency bands *δ* + *θ*.

The spectral profile of cortical EEG sources has been analyzed in patients with probable FTD compared with AD patients and HC [24]. The authors of this study differentiated 16 patients with AD from 19 patients with FTD using EEG band powers, coherence, dominant frequency, *α*-peak frequency, and cortical sources. Using logistic regression analysis, the best predictors of FTD and AD were defined in a model. These predictors included *δ* and *θ* activities together with high levels of visuospatial ability and episodic memory. Classification accuracy of the model was 93.3%. Therefore, the combination of qEEG and neuropsychological tests significantly contributes to classification accuracy and should be recommended for differential diagnoses of FTD and AD.

Caso et al. differentiated 39 AD from 39 FTD patients by means of power spectral analysis and standardized sLORETA within the *δ*, *θ*, *α*1, *α2*, *β*1, *β*2, and *β*3 frequency bands, achieving 49% sensitivity and 85% specificity [25]. As such, the sensitivity is at chance level. Both analyses revealed in AD patients higher expression of diffuse *δ*/*θ* and lower central/posterior fast frequency (from *α*1 to *β*2) bands compared to HC. Patients with FTD showed diffuse increased *θ* power compared with HC and lower *δ* compared to AD patients. Compared with FTD, AD patients showed diffuse higher *θ* power in the power spectrum and, by use of sLORETA, decreased *α*2 and *β*1 values in central/temporal regions. Again, we observe the relative increase of relevance of slower frequencies and decrease of faster frequencies.

**Figure 2:**
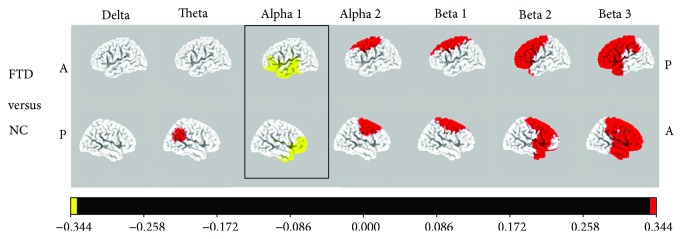
Comparison of current density images in Talairach space obtained by sLORETA for the FTD and control groups. Red areas correspond to significantly higher activity in the FTD group, and yellow areas correspond to significantly higher activity in the control group (*p* < 0.047, log-*F*-ratio threshold = 0.344). Legend: A = anterior; P = posterior; FTD = frontotemporal dementia; AD = Alzheimer's disease; NC = normal control. Reproduced with permission from Nishida et al. [26] in 2011.

Analyses of global field power, which is a measure of whole-brain electric field strength, together with EEG neuroimaging analyses with sLORETA, were performed in patients with mild stages of FTD and in HC [26]. In the global field power, significant group effects were observed in the *δ* (1.5–6.0 Hz), *α*1 (8.5–10.0 Hz), and *β*1 (12.5–18.0 Hz) bands. In sLORETA analysis, differences in activity were observed in the *α*1 band (HC > FTD) in the orbital frontal and temporal lobe, in the *δ* band (AD > HC) in widespread areas including the frontal lobe, and in the *β*1 band (FTD > AD) in the parietal lobe and sensorimotor area (Figure 2). As such, it does not seem that a specific brain region is relevant for the distinction of these groups.

Snaedal et al. first investigated the possibility of differentiating between 239 patients with AD, 52 patients with PDD or probable DLB (DLBPD), and 14 patients with FDT by qEEG [27]. The authors of this Icelandic study used *θ*, *α*2, and *β*1 coherences together with peak *α* frequency for classification. Using a support vector machine for classification, a good-to-excellent separation was found when differentiating cases of degenerative disorders from HC, but this was less so when the likelihood of comorbidity was high. The authors achieved 91% accuracy in differentiating AD from DLBPD, 93% for DLBPD-FTD, and 88% for AD-FTD. However, the accuracy of these statistical estimates must be interpreted with caution, given the very small sample of FTD patients. In general, studies involving FTD have to struggle with difficulties in recruiting participants, so that the importance of this study should not be undermined. Nevertheless, classification analysis requires adequate feature subset selection, especially in studies with long feature vectors such as in this study, where 1120 entries were taken into consideration. It is not clear whether the 10-fold cross-validation in this study with the genetic algorithm used a separate training, evaluation, and testing set.

**Figure 3:**
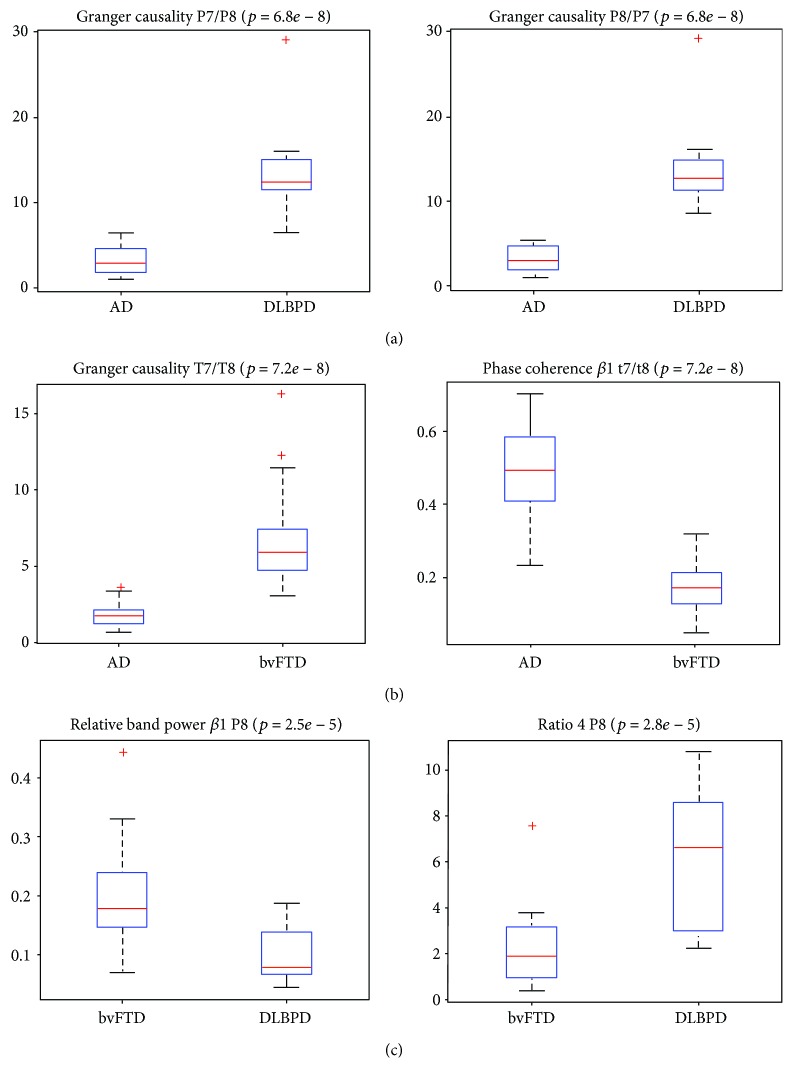
Boxplots of selected features: AD versus DLBPD: 23 of 25 features resulted in significant differences. The features with the lowest *p* values (*p* = 6.80e − 08) were the GC at P7/P8 and P8/P7 (a). Center frequency and relative band power *α* and *β*1 were higher in AD patients than in DLBPD patients at all sites with significantly different results. The opposite was true for automutual information, band ratios, relative band power *θ*, and cross-mutual information. AD versus bvFTD: 17 features resulted in significant differences with GC and phase coherence *β*1 reaching the lowest *p* value of 7.21*e* − 8 at T7/T8. GC was significantly higher in bvFTD patients than in AD patients while phase coherence was significantly higher in AD patients (b). Phase coherences *α* and *β*1 were significantly higher in AD patients than in bvFTD patients at all sites with significantly different results. The opposite was observed for coherence *β*1 and GC. bvFTD versus DLBPD: 12 features resulted in significant differences with relative band power *β*1 at P8 and ratio 4 at P8 having the lowest *p* value of 2.53*e* − 5 and 2.83*e* − 5, respectively. Automutual information and mutual information were higher in DLBPD patients than in bvFTD patients at all sites with significantly different results. The opposite was then evident for center frequency. Legend: DLBPD = PDD or probable DLB; bvFTD = behavioral variant of FTD; AD = Alzheimer's disease; GC = Granger causality. Reproduced with permission from Garn et al. [28] in 2017.

A recent study aimed at developing a classifier for differentiating probable AD from PDD or DLB and from bvFTD based on qEEG [28]. Twenty-five qEEG features characterizing frequency (spectral) properties, synchrony, and similarity of signals have been investigated. For analysis, scatter plots of these qEEG features versus MMSE scores were generated with linear regression lines with age and sex introduced as covariables. Twenty-three out of the 25 features were significantly different between AD and DLBPD; seventeen turned out to be significantly different for AD versus bvFTD, and 12 turned out to be significantly different for bvFTD versus DLBPD. The classification achieved an accuracy, sensitivity, and specificity of 100% using only the QEEG features of Granger causality (GC) and the ratio of *θ* and *β*1 band powers (Figure 3). These results suggest that classifiers trained with selected qEEG features can provide a valuable input in distinguishing among AD, DLB or PDD, and bvFTD patients. Comparing AD and bvFTD, conditional GC Fp1/Fp2 increases as the MMSE score decreases in AD patients and it decreases as MMSE score decreases in bvFTD patients. The opposite was observed for conditional GC O1/Fp1. A difference was observed for the feature partial coherence *α* at P7/P8 between bvFTD and DLBPD patients, where there was an increasing trend in DLBPD as the MMSE scores decreased and the opposite for bvFTD. GC, phase coherence *α*, phase coherence *β*1, and coherence *β*1 features at 29, 14, 16, and 18 sites or pairs of sites, respectively, were significantly different for the differentiation between AD and bvFTD.

### 3.3. EEG Has Been Used Also to Investigate Functional Connectivity in AD and FTD

Automutual information, mutual information, and center frequency at 5, 6, and 5 sites or pairs of sites, respectively, for differentiating bvFTD and DLBPD were found to be significant. This finding demonstrates that AD and DLBPD are most dissimilar based on the number of electrode sites. Comparing AD and bvFTD, it was observed that phase coherences *α* and *β*1 were higher in AD patients than in bvFTD patients. The opposite was observed for coherence *β*1 and GC. Automutual information and cross-mutual information were higher in DLBPD patients than in bvFTD patients, whereas center frequency is higher in bvFTD patients than in DLBPD patients. Yu et al. investigated functional connectivity and network topology in 69 AD patients, 48 bvFTD patients, and 64 individuals with subjective cognitive decline using resting-state EEG recordings [29]. Functional connectivity between all pairs of EEG channels was assessed using the phase lag index (PLI). Subsequently, the authors calculated PLI-weighted networks, from which minimum spanning trees (MSTs) were constructed. Finally, the hierarchical clustering organization of the MSTs has been investigated. Functional connectivity analysis showed frequency-dependent results: in the *δ* band, bvFTD showed the highest whole-brain PLI; in the *θ* band, the whole-brain PLI in AD was higher than that in bvFTD; and in the *α* band, AD showed lower whole-brain PLI compared with bvFTD and subjective cognitive decline. These findings suggest that frontal networks are selectively involved in bvFTD against the background of preserved global efficiency, whereas in AD parietal and occipital impairment of network organization is accompanied by global efficiency loss.

A more recent study relied on EEG signals and a novel information-sharing method to study resting-state connectivity in patients with bvFTD, AD, and HC [30]. Unlike AD patients, bvFTD patients showed a specific pattern of hypoconnectivity in midrange frontotemporal links. These functional connectivity abnormalities in bvFTD were observed with a low-density EEG setting (20 electrodes). Therefore, classification between bvFTD and AD patients was better when based on connectivity than on neuropsychological measures. Taken together, such findings underscore the relevance of EEG measures as potential biomarker signatures for clinical settings.

## 4. Discussion

Most studies on the differentiation among FTD, AD, DLB, or PDD, were done using SPECT, PET, or MRI. However, besides their expensiveness, these imaging techniques are not sufficient to provide information on the pathophysiological mechanisms of dementia, in particular in the early stages. We summarized in this narrative review the most relevant studies which aimed to distinguish FTD from AD and other dementias by using various types of EEG analyses.

It is well known that EEG activity can be influenced by the severity of dementia [24, 25, 27].

Indeed, the impairment of cortical neuronal networks related to cognitive functions is partially reflected by the abnormal mechanisms of cortical neural synchronization and by dysfunctional neuroplasticity of the neural transmission that generate resting EEG rhythms [31].

Since the likelihood of abnormal EEG findings seems to increase late in FTD compared to AD [15], Caso et al. aimed to evaluate whether also at an early stage of FTD EEG differences can be found in comparison to mild AD and HC, using a combined spectral and sLORETA approach. In AD patients compared to HC, EEG spectral analysis showed a significant occipital power increase within the *δ* band but a significant parietooccipital *α*1 and temporal *α*2 power decrease and widespread *β*1 and *β*2 power decrease [25]. Notably, these findings are consistent with those of many previous quantitative EEG studies [20, 21, 32–34]. On the other hand, the spectral pattern of EEG recordings in FTD patients did not significantly differ from HC except for a widespread increase of *θ* power, as previously reported [5, 35–37]. Compared with AD, FTD patients showed in the study of Caso et al. (differently from a previous work of Lindau et al. [24]) a decrease of *δ* power and higher *α*2 and *β*1 values over the posterior regions. Moreover, using classical spectral analysis the authors failed to find highly significant differences between FTD and AD in fast activities. sLORETA results were similar to those obtained by classical spectral analysis comparing AD patients with HC and FTD with HC. This is in agreement with previous studies using LORETA in AD [38–40]. Notably, *θ* band values seem to be more critical in differentiating HC from patients which are in a very early stage of mild AD [38]. Both spectral and sLORETA analysis for *α* activity in AD patients compared with HC were well preserved over the frontal areas. This finding may be explained by the so-called “anteriorization” of *α* rhythm in AD. In normal subjects, *α* generators are localized over the posterior regions of the brain while in AD the decrease of posterior *α* activity produces a shift to more anterior regions [17, 41, 42]. Comparing sLORETA results in the AD and FTD groups, a decreased *δ* power over the posterior regions was detected in FTD patients, in line with the findings of spectral analysis. Moreover, lower values within the posterior *α*2 and centrotemporal *β*1 bands were detected in AD compared with FTD patients. EEG cortical activity depends on a complex balance between cholinergic pathways and other neurotransmitters systems [43]. It is known that *α* rhythms are mainly modulated by thalamocortical interactions, which modulate the transmission of sensorimotor and cognitive information among subcortical and cortical pathways [44–46]. Therefore, it can be speculated that the magnitude reduction of fast cortical rhythms in mild AD is related to the impairment of cholinergic pathways, resulting in an abnormal increase of cortical excitation or disinhibition in the resting state. In FTD, the intracortical disconnection seems to be related to the neuronal loss in the frontal/frontotemporal area, alongside with the preserved cholinergic system. These characteristics may explain EEG differences in comparison to HC and AD patients. However, the results of Caso et al. [25] are only partially in agreement with the recent findings of Nishida et al. [26], showing a decrease of *α* band compared to HC and an increase of *β* band in comparison with AD in FTD patients. Moreover, no significant differences were found between AD and FTD in slow frequency bands or in the *α* band [26]. The different sample sizes of patients, and thus, the statistical power, of the two studies may explain this discrepancy. In addition, the different lengths of the analyzed EEG signals for each subject might also contribute to the differences between the two studies. Indeed, in the study of Nishida et al., only 40 seconds of EEG were analyzed in each participant. This weakly consistent spectral estimation might not be a fully representative interval and could provide a statistical correlation between cortical powers, sources of EEG rhythms, and cognitive functions of patients. As such, *α*1 power over frontal areas correlated positively with MMSE and Token Test scores, confirmed by both spectral and sLORETA analysis. It may be hypothesized that the intensity of *α*1 power changes in pathological aging is a function of the global cognitive level [47].

Even if the combination of sLORETA and spectral characterization seems to improve the sensitivity, the validity of spectral biomarkers for differentiation of single patients has not yet been confirmed. Nevertheless, the reviewed studies showed that both classical EEG spectral analysis and EEG source analysis were able to differentiate AD, FTD, and HC at group level. In fact, spectral analysis and sLORETA provided information that their combination can improve the characterization of EEG rhythmic activities in patients with the AD and FTD group. Further studies with larger sample sizes and considering the combination of spectral and sLORETA analysis with imaging techniques, such as brain MRI and/or PET/SPECT, could be useful in order to improve the classification of single subjects.

One additional problem in several of the presented EEG studies should be mentioned. Machine learning techniques have emerged over the past two decades and are now an integral part of clinical neuroscientific research. However, older studies have failed to clearly separate the training from the testing data, so that most of the older results include a considerable amount of overfitting. Today, the standard is the separation of the data into three sets: training, evaluation, and testing sets. Without this clear separation, the reported results must be interpreted with caution. As a rough estimate, a result of 100% accuracy with only two sets could be 80% or less, when overfitting is avoided by clear separation into three sets.

Slowing of the EEG frequency spectrum, which has long been known to be a hallmark in dementia, was confirmed to represent one of the two most significant features for differential diagnosis. This is well in line with the results of previous studies [48]. Interestingly, qEEG features correlated with the severity of disease measured by MMSE scores.

EEG variability in DLB may be associated with the fluctuating cognition seen in these patients. This might have clinical implications for the diagnosis of DLB. Despite that modern techniques of qEEG analysis can be quite sophisticated, spectral EEG analysis is simple enough to be incorporated into clinical software, which would pave the way for the implementation of qEEG into clinical practice.

It is necessary to conduct further studies with larger sample sizes in order to confirm the reviewed findings. It is also of interest to validate the hypotheses by adding further comparisons to HC, although this would not affect the differentiation among AD, DLBPD, and bvFTD patients with MMSE scores less than 30. However, the comparison to HC would establish a more solid knowledge about the baseline, and thus, allow drafting the course of pathological changes from healthy aging, to early and more severe stages of the examined diseases.

Notably, genetic factors are often associated with FTD; in particular, the microtubule-associated protein tau (MAPT), the chromosome 9 open reading frame 72 (c9orf72), and granulin/progranulin have been identified as common FTD genes. Future studies should also take into account genetic differences in a sample population. Neurophysiological techniques are able to detect distinct and peculiar abnormalities associated with different genetic features that are the expression of precise FTD phenotypes. Indeed, in healthy human subjects EEG measures were able to identify some differences in genetically different groups [49].

One of the drawbacks of most studies is that they rely on clinical features instead of definite diagnoses. Indeed, the most EEG studies did not have any postmortem confirmation. The diagnoses were clinically based on MRI/CT and/or SPECT/PET imaging, in order to avoid the inclusion of phenocopies in the FTD sample. However, Knopman et al. [50] demonstrated that an accurate FTD antemortem diagnosis is possible by combining clinical, neuropsychological, and imaging features (MRI scan), giving a sensitivity of 85% and a specificity of 99%. Moreover, in McNeill's report [51] the percentages of correct diagnoses using the association of SPECT results and clinical data were 92% for FTD and 90.3% for AD patients.

In future studies, the possible impact of combining EEG with other neurophysiological techniques, such as transcranial magnetic stimulation (TMS), should also be carefully addressed. TMS-EEG has been demonstrated to be a suitable, reliable, and affordable tool for detecting changes in cortical excitability, connectivity, and functional synchronization of EEG activity both in normal aging [52, 53] and in AD [54]. The analysis of TMS-evoked oscillations could possibly allow detecting subtle and area-specific alterations of natural oscillatory activities [55] with good sensitivity and specificity for different types of dementia.

Some systems do already allow for a quantitative spectral analysis, but further processing of the signal is highly warranted for clinical decision making. Modern qEEG scoring systems such as, for instance the dementia index SIGLA (http://www.mentiscura.com), give an exact answer to the question whether a patient suffers from DLB or whether he will develop AD. We anticipate that such systems will enter the clinical arena within the next 10 years and ease the use of qEEG for clinicians tremendously.

In conclusion, application of EEG techniques in neurodegenerative diseases has provided important pathophysiological insights, leading to the development of pathogenic and diagnostic biomarkers that could be used in the clinical setting and therapeutic trials.
